# Publisher Correction: Overcoming the coherence time barrier in quantum machine learning on temporal data

**DOI:** 10.1038/s41467-024-53536-3

**Published:** 2024-10-23

**Authors:** Fangjun Hu, Saeed A. Khan, Nicholas T. Bronn, Gerasimos Angelatos, Graham E. Rowlands, Guilhem J. Ribeill, Hakan E. Türeci

**Affiliations:** 1 Department of Electrical and Computer Engineering, Princeton University, Princeton, NJ USA; 2 IBM Quantum, IBM T.J. Watson Research Center, Yorktown Heights, NY USA; 3 RTX BBN Technologies, Cambridge, MA USA

**Keywords:** Quantum information, Information theory and computation

Correction to: *Nature Communications* 10.1038/s41467-024-51162-7, published online 30 August 2024

In the original version of this Article, the following errors were present:

- The bibliography was missing a reference, “Martínez-Peña, R. & Ortega, J.-P. Quantum reservoir computing in finite dimensions. *Phys. Rev. E* **107**, 035306 (2023)” which should have appeared at number [53].
- Because of the above, two mentions of ref. 53—and in particular the ones in “see “Methods” subsection “The quantum Volterra theory and analysis of NISQRC” and also ref. 53” and within the “Results” section, “A similar result for quantum reservoirs characterized by quantum channels can also be found from Theorem 2 in ref. 53” within the “Methods” section, were incorrectly referring to a different work, that is “Suzuki, Y., Gao, Q., Pradel, K. C., Yasuoka, K. & Yamamoto, N. Natural quantum reservoir computing for temporal information processing. *Sci. Rep*. **12**, 1353 (2022).”

This has now been corrected, and the rest of the references are renumbered.

