# Supplementary material for: Overcoming the coherence time barrier in quantum machine learning on temporal data
Source: Nat Commun. 2024 Aug 30;15:7491. doi: 10.1038/s41467-024-51162-7 (PMC11364873; doi:10.1038/s41467-024-51162-7)
Supplement: Supplementary file 1 — Supplementary Information [file 41467_2024_51162_MOESM1_ESM.pdf]

SUPPLEMENTARY INFORMATION

Supplementary Information 1

1 Details of the NISQRC architecture 1
1 The NISQRC algorithm . . . 1

2 Quantum dynamics under NISQRC – Role of repeated evolution, measurement, and reset 2
1 Quantum dynamics under measurement without subsequent qubit reset . . . 2
2 Quantum dynamics under measurement and reset . . . 4

3 Deriving the NISQRC quantum I/O map 6
1 Technique of u-expansion and $\mathcal{R}_k$ and $\mathcal{P}_k$ superoperators . . . 6
2 $\mathcal{R}_k$ and $\mathcal{P}_k$ for linear Hamiltonian encoding scheme by regrouping the BCH formula . . . 7
3 Functional I/O map: time-invariance and Volterra kernels . . . 8
4 u-expansion and Volterra kernels for dissipative quantum systems . . . 9

4 Fading memory modes 10

5 Relation between functional-independence and Jacobian rank 12

6 Channel equalization: background and training details 13

7 IBM Device simulations as a function of qubit coherence times 14

8 IBM Device experiments under controlled delays 14

9 Lists of device parameters 15

Supplementary References 17

Supplementary Note 1: Details of the NISQRC architecture

1. The NISQRC algorithm

The underlying dynamical system we analyze in this article consists of $L = M + R$ qubits, with M qubits serving as memory qubits and R qubits serving as readout qubits. The evolution is governed by a Hamiltonian that is linearly parameterized by a one-dimensional variable $u \in [-1, 1]$ (serving as input):

$$\hat{H}(u) = \hat{H}_0 + u \cdot \hat{H}_1. \tag{1}$$

We choose a form of $\hat{H}_0$ and $\hat{H}_1$ that can be implemented in a quantum annealing system or analog quantum simulator in a hardware-efficient way: $\hat{H}_0 = \sum_{\langle i,i' \rangle} J_{i,i'} \hat{\sigma}_i^z \hat{\sigma}_{i'}^z + \sum_{i=1}^L \eta_i^x \hat{\sigma}_i^x$ and $\hat{H}_1 = \sum_{i=1}^L \eta_i^z \hat{\sigma}_i^z$ The coupling strength $J_{i,i'}$, transverse x-field strength $\eta_i^x = \eta^x + \varepsilon_i^x$ and longitudinal z-drive strength $\eta_i^z = \eta^z + \varepsilon_i^z$ are pre-selected via randomness: $J_{i,i'} \sim \mathrm{Unif}[0, J_{\max}]$, $\varepsilon_i^x \sim \varepsilon_{\mathrm{rms}}^x \times \mathcal{N}(0,1)$ and $\varepsilon_i^z \sim \varepsilon_{\mathrm{rms}}^z \times \mathcal{N}(0,1)$. One thing that needs to be emphasized is that the encoding scheme Supplementary Equation 1 is general enough such that encoding Eq. (7) is merely an illustrative example. A variety of $\hat{H}_0, \hat{H}_1$ can be employed as long as they are resource-efficiently realized in a physical platform.

In theory, the domain $\mathbb{Z}$ of n is infinite. However in practical experiments, it is impossible to feed an input sequence from infinite past $n = -\infty$ to infinite future $n = \infty$. Thus we cutoff infinity of time-step index into $n \in [N] \equiv \{1, 2, \cdots, N\}$. As

a summary, now we have a sequence of reservoir recurrent units, each of which is characterized by an underlying Hamiltonian $H(u_n)$ for all $n \in [N]$, and step evolution duration τ.

As what we will prove in Supplementary Note 2, since calculating readout feature functions $\{x_j(n)\}_{j\in[K]}$ can be done by taking $x_j(n) = \mathrm{Tr}\left(\hat{M}_j \hat{\rho}_n^{\mathsf{MR}}\right)$ where the effective density matrix is $\hat{\rho}_n^{\mathsf{MR}} = \mathcal{U}(u_n)\left(\left(\mathcal{C}(u_{n-1})\cdots\mathcal{C}(u_1)\hat{\rho}_0^{\mathsf{M}}\right) \otimes |0\rangle\langle 0|^{\otimes R}\right)$, the full dynamics of NISQRC can also be written into set of recurrent equations

$$\begin{cases} \hat{\rho}_n^{\mathsf{MR}} = \mathcal{U}(u_n)\left(\hat{\rho}_{n-1}^{\mathsf{M}} \otimes |0\rangle\langle 0|^{\otimes R}\right), \\ \mathbf{x}(n) = \{x_j(n)\}_{j\in[K]} = \{\mathrm{Tr}(\hat{M}_j \hat{\rho}_n^{\mathsf{MR}})\}_{j\in[K]}, \\ y_n = \mathbf{w}\cdot\mathbf{x}(n). \end{cases} \tag{2}$$

This algorithm induces a functional $\mathcal{F} : \mathbf{u} \mapsto \mathbf{y}$, where $\mathbf{y}(n) = y_n$. We define an observable

$$\hat{M}_{\mathbf{w}} \equiv \sum_{j=0}^{K-1} w_j \hat{M}_j, \tag{3}$$

and therefore $y_n = \mathrm{Tr}(\hat{M}_{\mathbf{w}} \hat{\rho}_n^{\mathsf{MR}})$ which affords a great deal of convenience in our notation.

The readout features $x_j(n)$ are nothing but the respective probabilities of measuring $\mathbf{b}_j$ at the n-th time step, and we call this readout scheme the *probability representation* [1]. In the literature, the readout features are alternatively chosen to be the quantum spin moments. In this *moment representation*, $\mathcal{O}'_R = \{\hat{M}_j | \hat{M}_j = I^{\otimes M} \otimes \bigotimes_{i=M+1}^{L} \hat{\sigma}_i\}$ where each $\hat{\sigma}_i \in \{\hat{I}, \hat{\sigma}^z\}$. These two different representations can be related by a Walsh-Hadamard transformation [1].

Supplementary Note 2: Quantum dynamics under NISQRC – Role of repeated evolution, measurement, and reset

1. Quantum dynamics under measurement without subsequent qubit reset

For simplicity, we first consider a QRC with $M = 1$ memory qubit and $R = 1$ readout qubit (namely $L = M + R = 2$). Furthermore, we consider $\hat{\sigma}^z$ measurement of the readout qubit at each time step n of the framework. The measurement Kraus operators introduced in the main text then take the specific form

$$\hat{P}_i = \hat{I} \otimes |i\rangle\langle i|\,. \tag{4}$$

The corresponding observable M can be written as $\hat{M} = \sum_{i=0}^{1} i\hat{P}_i = \hat{I} \otimes |1\rangle\langle 1|$, which measures the probability of the single readout qubit being in excited state.

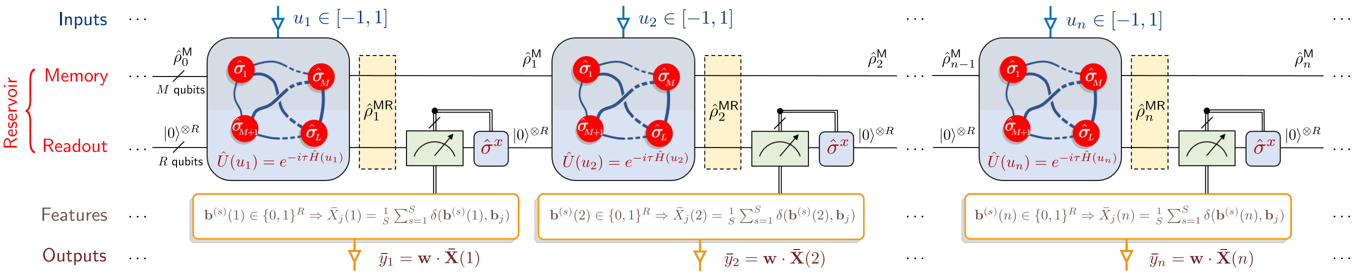

Supplementary Figure 1. NISQRC architecture to generate a functional map $\mathcal{F} : \mathbf{u} \mapsto \mathbf{y}$ by using a qubit-based quantum system. The input function can be written as a time-discrete sequence $\mathbf{u} = \{u_{-\infty}, \cdots, u_{-1}, u_0, u_1, \cdots, u_\infty\}$, which is encoded in the quantum system at every time step n via a fixed encoding scheme, here shown as a Hamiltonian encoding $\hat{H}(u_n)$. Measured features $\bar{X}_j(n)$ are constructed from finite samples S under a specified measurement scheme at each time step (for example probabilities of measured bit-strings $\mathbf{b}^{(s)}(n)$ under computational basis measurement). The output function $\mathbf{y} = \{y_{-\infty}, \cdots, y_{-1}, y_0, y_1, \cdots, y_\infty\}$ is constructed from these finitely-sampled measured features. The goal of the trained functional $\mathcal{F}$ is to approximate a desired functional $\mathcal{F}^\star : \mathbf{u} \mapsto \mathbf{y}^\star$, where $\mathbf{y}^\star = \{y^\star_{-\infty}, \cdots, y^\star_{-1}, y^\star_0, y^\star_1, \cdots, y^\star_\infty\}$, so that under the same input $\mathbf{u}$, $y^\star_n \approx y_n \;\forall\, n$ with as little error as possible.

The NISQRC framework then involves a continuous pipeline of evolution under a quite arbitrary superoperator $\mathcal{U}(n)$ (not restricted to the linearly parameterized Hamiltonian form we consider in the main text), followed by measurement, repeated until all inputs $\{u_n\}$ have been processed by the QRC. The inclusion of measurement with stochastic outcomes interleaved with evolution steps, as opposed to at the final step, makes our knowledge of the QRC state conditional on the entire measurement history. For example, starting from the initial state $\hat{\rho}_0^{\mathsf{MR}}$ and evolving under $\mathcal{U}_1$ at time step $n=1$, the subsequent measurement yields a measurement outcome $X_n = i_n$, where $i_n \in \{0,1\}$ for a single readout qubit. The post-measurement state $\hat{\rho}_1^{\mathsf{MR,cond}}$ is then conditioned on the measurement result at time step $n=1$, as indicated by the superscript cond. For an arbitrary time step n, this conditioning thus extends to the entire measurement history $\{X_1, X_2, \ldots, X_{n-1}\}$. The entire pipeline can be viewed schematically as below:

$$
\begin{array}{ccccccccc}
\hat{\rho}_0^{\mathsf{MR}} & \xrightarrow{\mathcal{U}_1,\hat{P}_{i_1}} & \hat{\rho}_1^{\mathsf{MR,cond}} & \xrightarrow{\mathcal{U}_2,\hat{P}_{i_2}} & \hat{\rho}_2^{\mathsf{MR,cond}} \cdots & \xrightarrow{\mathcal{U}_n,\hat{P}_{i_n}} & \hat{\rho}_n^{\mathsf{MR,cond}} \cdots \\
 & & \Downarrow & & \Downarrow & & \Downarrow \\
 & & X_1 & & X_2 & & X_n
\end{array}
\tag{5}
$$

It is not hard to show that this process is equivalent to the quantum non-demolition scheme proposed in Ref. [2].

In practice, we are often interested not in the result of a single shot, but of the ensemble average computed over many shots; in the limit of infinite-sampling, this defines the readout features $x(n)$ computed via ensemble averages over an infinite number of repeated shots of their stochastic conditional counterparts X_n:

$$
x(n) = \mathbb{E}[X_n]. \tag{6}
$$

Computing this expectation using individual measurement shots would be the standard approach in any experimental NISQRC realization, but is prohibitively expensive for this analysis. This is not least because of the dependence of X_n at any time step n on the entire measurement history $\{X_1, X_2, \ldots, X_{n-1}\}$, a complexity that scales very unfavourably with QRC size and the total number of time steps N. Instead, we show that the expectation can be efficiently evaluated - crucially, accounting for the conditional dynamics due to interleaved measurements - to yield a simplified expression for the infinitely-sampled readout features in terms of an effective, ensemble-averaged density matrix $\hat{\rho}_n^{\mathsf{MR}}$, namely $x(n) = \mathrm{Tr}(\hat{M}\hat{\rho}_n^{\mathsf{MR}})$.

To proceed, we note that, by mathematical induction, the conditional state with associated measurement record $\{X_1 = i_1, \cdots, X_{n-1} = i_{n-1}\}$ is

$$
\hat{\rho}_n^{\mathsf{MR,cond}} = \frac{\mathcal{U}_n\left(\hat{P}_{i_{n-1}} \cdots \mathcal{U}_2\left(\hat{P}_{i_1}\left(\mathcal{U}_1\hat{\rho}_0^{\mathsf{MR}}\right)\hat{P}_{i_1}^{\dagger}\right)\cdots \hat{P}_{i_{n-1}}^{\dagger}\right)}{\mathrm{Tr}\left(\hat{P}_{i_{n-1}} \cdots \mathcal{U}_2\left(\hat{P}_{i_1}\left(\mathcal{U}_1\hat{\rho}_0^{\mathsf{MR}}\right)\hat{P}_{i_1}^{\dagger}\right)\cdots \hat{P}_{i_{n-1}}^{\dagger}\right)}, \tag{7}
$$

while the probability of obtaining this measurement record is simply

$$
\Pr[X_1 = i_1, \cdots, X_{n-1} = i_{n-1}] = \mathrm{Tr}\left(\hat{P}_{i_{n-1}} \cdots \mathcal{U}_2\left(\hat{P}_{i_1}\left(\mathcal{U}_1\hat{\rho}_0^{\mathsf{MR}}\right)\hat{P}_{i_1}^{\dagger}\right)\cdots \hat{P}_{i_{n-1}}^{\dagger}\right). \tag{8}
$$

In order to further simplify this expression, we observe the following identity for any $\hat{A} \in \mathbb{C}^{4\times 4}$, which can be verified by direct computation

$$
\sum_{i=0,1} \hat{P}_i \hat{A} \hat{P}_i^{\dagger} = (\hat{\mathbf{1}} \otimes \hat{I}) \circ \hat{A} \tag{9}
$$

where the matrices $\hat{I} = \begin{pmatrix} 1 & 0 \\ 0 & 1 \end{pmatrix}$ and $\hat{\mathbf{1}} = \begin{pmatrix} 1 & 1 \\ 1 & 1 \end{pmatrix}$, and the notation $\circ$ represents the *Hadamard product* (element-wise product): $(\hat{A} \circ \hat{B})_{ij} = A_{ij}B_{ij}$. Supplementary Equation 9 enables us to introduce the *measurement-induced decoherence superoperator* $\mathcal{M}$:

$$
\mathcal{M}\hat{\rho}^{\mathsf{MR}} = (\hat{\mathbf{1}} \otimes \hat{I}) \circ \hat{\rho}^{\mathsf{MR}}. \tag{10}
$$

Therefore, according to Supplementary Equation 8, the unconditional expectation $\mathbb{E}[X_n] = \sum_{i_1,\cdots,i_n} i_n \Pr[X_1 = i_1, \cdots, X_n = i_n]$ of the random variable X_n can be computed by contraction:

$$
x(n) = \mathbb{E}[X_n] = \sum_{i_1,\cdots,i_n} i_n \mathrm{Tr}\left(\hat{P}_{i_n}\mathcal{U}_n\left(\cdots \mathcal{U}_2\left(\hat{P}_{i_1}\left(\mathcal{U}_1\hat{\rho}_0^{\mathsf{MR}}\right)\hat{P}_{i_1}^{\dagger}\right)\cdots\right)\hat{P}_{i_n}^{\dagger}\right) = \mathrm{Tr}\left(\hat{M}\left(\mathcal{U}_n\mathcal{M}\mathcal{U}_{n-1}\cdots\mathcal{U}_2\mathcal{M}\mathcal{U}_1\hat{\rho}_0^{\mathsf{MR}}\right)\right). \tag{11}
$$

where we used Supplementary Equation 10 and $\hat{M} = \sum_{i_n} i_n \hat{P}_{i_n}$. This expression naturally leads to the identification of the term in square brackets as the effective density matrix at time step n, $\hat{\rho}_n^{\mathsf{MR}} = \mathcal{U}_n \mathcal{M} \cdots \mathcal{U}_2 \mathcal{M} \mathcal{U}_1 \hat{\rho}_0^{\mathsf{MR}}$, such that computing the trace with respect to this density matrix provides any readout feature at time step n in the infinite sampling limit, $x(n) = \mathrm{Tr}(\hat{M} \hat{\rho}_n^{\mathsf{MR}})$. The generalization to a QRC with $L = M + R \geq 3$ and input sequence $\{u_n\}$ is now straightforward: $\mathcal{U}_n$ is replaced with $\mathcal{U}(u_n)$, while the measurement-induced decoherence superoperator $\mathcal{M}$ generalizes to:

$$\mathcal{M}\hat{\rho}^{\mathsf{MR}} = \left(\hat{\mathbf{1}}^{\otimes M} \otimes \hat{I}^{\otimes R}\right) \circ \hat{\rho}^{\mathsf{MR}}. \tag{12}$$

With these changes, the effective density matrix at time step n for the NISQRC framework without reset is given by

$$\hat{\rho}_n^{\mathsf{MR}} = \mathcal{U}(u_n) \mathcal{M} \mathcal{U}(u_{n-1}) \cdots \mathcal{U}(u_2) \mathcal{M} \mathcal{U}(u_1) \hat{\rho}_0^{\mathsf{MR}}. \tag{13}$$

Note that $\hat{\rho}_n^{\mathsf{MR}}$ accounts for both any time-dependent unitary dynamics via $\mathcal{U}_n$, as well as the role of repeated measurements via recurrent applications of $\mathcal{M}$.

a. *Thermalization induced by repeated measurements without reset*

We need to point out that even if the circuits have similar structures to those used in measurement-induced phase transition [3]: at step n associated with unitary evolution $\mathcal{U}_n$, qubits indexed by a random subset $\mathcal{I}_n \subseteq [L]$ will be measured. In this scenario, the effective state evolution is similar $\hat{\rho}_n^{\mathsf{MR}} = \mathcal{U}_n \mathcal{M}_{\mathcal{I}_{n-1}} \cdots \mathcal{U}_2 \mathcal{M}_{\mathcal{I}_1} \mathcal{U}_1 \hat{\rho}_0^{\mathsf{MR}}$, the only difference is that measurement-induced decoherence superoperator $\mathcal{M}_n$ now is no longer a time-independent map

$$\mathcal{M}_{\mathcal{I}_n} \hat{\rho}^{\mathsf{MR}} = \left(\bigotimes_{i=1}^{L} \hat{E}_i\right) \circ \hat{\rho}^{\mathsf{MR}}, \quad \hat{E}_i = \begin{cases} \hat{I}, & \text{if } i \in \mathcal{I}_n, \\ \hat{\mathbf{1}}, & \text{if } i \notin \mathcal{I}_n. \end{cases} \tag{14}$$

For any overall state $\hat{\rho}^{\mathsf{MR}}$, the Frobenius distance $\left\|\hat{\rho}^{\mathsf{MR}} - \frac{I^{\otimes L}}{2^L}\right\|_F^2$ will never increase after either unitary evolution $\mathcal{U}$ or measurement $\mathcal{M}_{\mathcal{I}}$:

$$\left\|\mathcal{U}\hat{\rho}^{\mathsf{MR}} - \frac{\hat{I}^{\otimes L}}{2^L}\right\|_F^2 = \left\|\mathcal{U}\left(\hat{\rho}^{\mathsf{MR}} - \frac{\hat{I}^{\otimes L}}{2^L}\right)\right\|_F^2 = \left\|\hat{\rho}^{\mathsf{MR}} - \frac{\hat{I}^{\otimes L}}{2^L}\right\|_F^2, \tag{15}$$

$$\left\|\mathcal{M}_{\mathcal{I}}\hat{\rho}^{\mathsf{MR}} - \frac{\hat{I}^{\otimes L}}{2^L}\right\|_F^2 = \left\|\mathcal{M}_{\mathcal{I}}\left(\hat{\rho}^{\mathsf{MR}} - \frac{\hat{I}^{\otimes L}}{2^L}\right)\right\|_F^2 \leq \left\|\hat{\rho}^{\mathsf{MR}} - \frac{\hat{I}^{\otimes L}}{2^L}\right\|_F^2, \tag{16}$$

where the proof employs that fully mixed state $\frac{\hat{I}^{\otimes L}}{2^L}$ is the simultaneous fixed point of $\mathcal{U}$ and $\mathcal{M}_{\mathcal{I}}$ (equivalently, both maps are *unital* CPTP map). The non-increasing purity implies that

$$\lim_{n\to\infty} \hat{\rho}_n^{\mathsf{MR}} = \frac{\hat{I}^{\otimes L}}{2^L}. \tag{17}$$

The final QRC state therefore has no memory of the initial state $\hat{\rho}_0^{\mathsf{MR}}$. As a result, in previous works [4] this type of evolution has been employed to equip QRCs with the fading memory property. However, note that the final state is also entirely independent of the input $u(n)$, which renders it incapable of performing any useful computations on this input. Hence input-dependent unitary evolution combined with readout only does not yield a useful QRC. We show next how a simple modification of the measurement protocol can allow fading memory without yielding a trivial I/O map.

2. Quantum dynamics under measurement and reset

For notational simplicity, we once again analyze a system with $M = 1$ memory qubit and $R = 1$ readout qubit (namely $L = M + R = 2$). We apply Pauli z measurement on the readout qubit at each QRC step, the corresponding observable is $\hat{M} = \hat{I} \otimes |1\rangle\langle 1|$. Since now we apply the conditional reset. The measurement process is described by a POVM measurement ($i = 0, 1$):

$$\hat{K}_i = \hat{I} \otimes |0\rangle\langle i|, \tag{18}$$

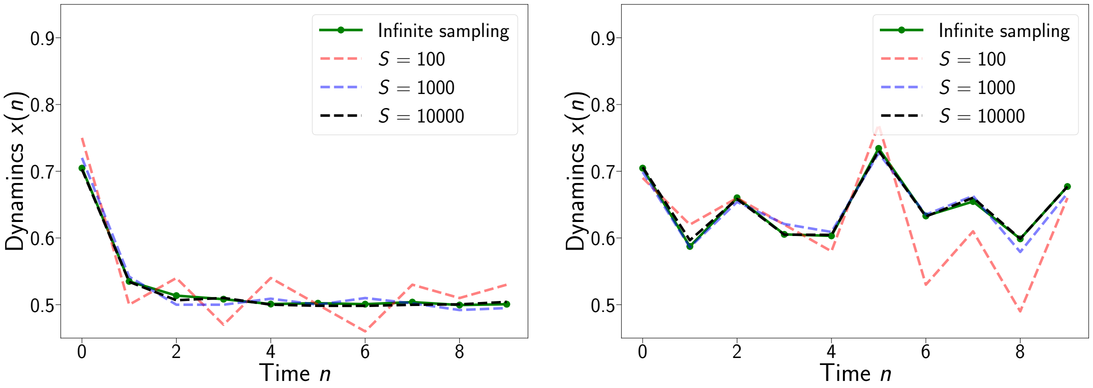

Supplementary Figure 2. NISQRC readout features for a $(2+1)$-qubit QRC, under both finite sampling (dashed lines) and infinite sampling (solid line and dots). The hyperparameters are $J_{\max} = \eta^x = \varepsilon^x_{\rm rms} = \eta^z = \varepsilon^z_{\rm rms} = 1$ in units of $1/\tau$. (Left) Without reset. (Right) With reset. In both cases, with increasing shots, the finitely-sampled readout features become closer to the black dashed features under infinite shots, as expected. However, without reset the readout features approach trivial values dictated by the effective density matrix of Supplementary Equation 17 as n increases.

and thus when overall state $\hat{\rho}^{\mathsf{MR}}$ is measured, the post-measurement state should be $\hat{K}_i \hat{\rho}^{\mathsf{MR}} \hat{K}_i^\dagger$ if the random readout index is i. These two POVMs satisfy the completeness relation:

$$\sum_{i=0,1} \hat{K}_i^\dagger \hat{K}_i = \sum_{i=0,1} \hat{I} \otimes |i\rangle\langle i| = \hat{I} \otimes \hat{I}. \tag{19}$$

The NISQRC pipeline including reset can now be viewed schematically as:

$$\begin{array}{ccccccccc} \hat{\rho}_0^{\mathsf{MR}} & \xrightarrow{\mathcal{U}_1, \hat{K}_{i_1}} & \hat{\rho}_1^{\mathsf{MR,cond}} & \xrightarrow{\mathcal{U}_2, \hat{K}_{i_2}} & \hat{\rho}_2^{\mathsf{MR,cond}} & \cdots & \xrightarrow{\mathcal{U}_n, \hat{K}_{i_n}} & \hat{\rho}_n^{\mathsf{MR,cond}} & \cdots \\ & & \Downarrow & & \Downarrow & & & \Downarrow & \\ & & X_1 & & X_2 & & & X_n & \end{array} \tag{20}$$

Proceeding as before, the conditional state with associated measurement record $\{X_1 = i_1, \cdots, X_{n-1} = i_{n-1}\}$ is

$$\hat{\rho}_n^{\mathsf{MR,cond}} = \frac{\mathcal{U}_n\left(\hat{K}_{i_{n-1}} \cdots \mathcal{U}_2\left(\hat{K}_{i_1}\left(\mathcal{U}_1 \hat{\rho}_0^{\mathsf{MR}}\right)\hat{K}_{i_1}^\dagger\right)\cdots \hat{K}_{i_{n-1}}^\dagger\right)}{\mathrm{Tr}\left(\hat{K}_{i_{n-1}} \cdots \mathcal{U}_2\left(\hat{K}_{i_1}\left(\mathcal{U}_1 \hat{\rho}_0^{\mathsf{MR}}\right)\hat{K}_{i_1}^\dagger\right)\cdots \hat{K}_{i_{n-1}}^\dagger\right)}, \tag{21}$$

and the probability of obtaining this measurement record $\{X_1 = i_1, \cdots, X_n = i_n\}$ is

$$\Pr[X_1 = i_1, \cdots, X_n = i_n] = \mathrm{Tr}\left(\hat{K}_{i_n}\mathcal{U}_n\left(\hat{K}_{i_{n-1}}\mathcal{U}_{n-1}\left(\cdots \hat{K}_{i_1}\left(\mathcal{U}_1 \hat{\rho}_0^{\mathsf{MR}}\right)\hat{K}_{i_1}^\dagger \cdots\right)\hat{K}_{i_{n-1}}\right)\hat{K}_{i_n}^\dagger\right). \tag{22}$$

which are analogous to the previous results with the replacement $\hat{P}_{i_n} \to \hat{K}_{i_n}$. Similar to Supplementary Equation 9, we can verify that for any $\hat{A} \in \mathbb{C}^{4\times 4}$,

$$\sum_{i=0,1} \hat{K}_i \hat{A} \hat{K}_i^\dagger = \mathrm{Tr}_{\mathsf{R}}(\hat{A}) \otimes |0\rangle\langle 0|\,. \tag{23}$$

For a quantum reservoir, we let $\mathcal{U}_n = \mathcal{U}(u_n)$. A similar contraction as Supplementary Equation 11

$$\sum_i \hat{K}_i \mathcal{U}(\hat{\rho}^{\mathsf{M}} \otimes |0\rangle\langle 0|)\hat{K}_i^\dagger = \mathrm{Tr}_{\mathsf{R}}(\mathcal{U}(\hat{\rho}^{\mathsf{M}} \otimes |0\rangle\langle 0|)) \otimes |0\rangle\langle 0| \tag{24}$$

gives the effective state evolution $x(n) = \mathrm{Tr}(\hat{M}\hat{\rho}_n^{\mathsf{MR}})$ where $\hat{\rho}_n^{\mathsf{MR}} = \mathcal{U}_n\left(\left(\mathcal{C}_{n-1}\cdots\mathcal{C}_1\hat{\rho}_0^{\mathsf{M}}\right)\otimes|0\rangle\langle 0|\right)$ and $\mathcal{C}_n\rho^{\mathsf{M}} = \mathrm{Tr}_{\mathsf{R}}\left(\mathcal{U}_n\left(\hat{\rho}^{\mathsf{M}} \otimes |0\rangle\langle 0|\right)\right)$.

Also, for more general $M > 1$ and $R > 1$ we used in the main text, we can still introduce the effective density matrices $\hat{\rho}_n^{\mathsf{MR}}$ in NISQRC having the same expression

$$\hat{\rho}_n^{\mathsf{MR}} = \mathcal{U}(u_n)\left(\left(\mathcal{C}(u_{n-1})\cdots\mathcal{C}(u_1)\hat{\rho}_0^{\mathsf{M}}\right)\otimes|0\rangle\langle 0|^{\otimes R}\right). \tag{25}$$

where $\mathcal{C}(u)\hat{\rho}^{\mathsf{M}} = \mathrm{Tr}_{\mathsf{R}}\left(\mathcal{U}(u)\left(\hat{\rho}_0^{\mathsf{M}}\otimes|0\rangle\langle 0|^{\otimes R}\right)\right)$. Hence, we finish deriving the expression of $\hat{\rho}_n^{\mathsf{MR}}$.

Supplementary Note 3: Deriving the NISQRC quantum I/O map

In this Supplementary Note, we will derive the I/O map of the NISQRC framework, ultimately arriving at the results presented in Eq. (1) of the main text.

1. Technique of u-expansion and $\mathcal{R}_k$ and $\mathcal{P}_k$ superoperators

In Supplementary Note 2, we have obtained concise formula Supplementary Equation 25 for evaluating the infinitely-sampled readout features $x_j(n)$ under a general superoperator $\mathcal{U}(n)$ and a simple quantum measurement and reset scheme. However, the explicit dependence of these readout features on the input $u(n)$ - which defines the I/O map implemented by the NISQRC scheme - is not yet apparent.

Uncovering this dependence requires addressing two complex, and in our framework, related issues. First, the I/O map is generally nonlinear in the input space. For example, in the Hamiltonian model we consider in main text, even if both the Hamiltonian encoding $\hat{H}(u) = \hat{H}_0 + u\cdot\hat{H}_1$ in Supplementary Equation 1 and readouts $\langle\hat{M}_{\mathbf{w}}\rangle_{\hat{\rho}_n^{\mathsf{MR}}}$ are linear, the evolution defined by $\hat{U}(u) = e^{-i\tau\hat{H}(u)}$ will clearly lead to a nonlinear dependence on the inputs at every time step. Secondly, the map also extends over past inputs: the NISQRC framework has memory. The dependence on past input history must be extricated by unraveling the recurrent structure of, for example, Supplementary Equation 25, necessitated by the multi-step nature of NISQRC for temporal data processing. We will show that both these complications are addressable within a unified framework using a Volterra series description.

The key theoretical tool we employ to achieve this is referred to as the *u-expansion*: an expansion of the superoperators governing dynamics in the NISQRC framework, including measurement and reset, in powers of the input u. More precisely, we wish to expand the superoperators $\mathcal{U}(u)$ and $\mathcal{C}(u)$ in terms of the monomial u^k:

$$\mathcal{U}(u)\hat{\rho}^{\mathsf{MR}} = \sum_{k=0}^{\infty} u^k\mathcal{R}_k\hat{\rho}^{\mathsf{MR}}, \tag{26}$$

$$\mathcal{C}(u)\hat{\rho}^{\mathsf{M}} = \sum_{k=0}^{\infty} u^k\mathcal{P}_k\hat{\rho}^{\mathsf{M}}. \tag{27}$$

for some superoperators $\{\mathcal{R}_k\}$, $\{\mathcal{P}_k\}$ respectively.

Regardless of the exact expression of u-expansion of the other dynamical superoperator, $\mathcal{C}(u)\hat{\rho}^{\mathsf{M}}$, the relationship between $\mathcal{U}(u)$ and $\mathcal{C}(u)$ means that the u-expansion of the latter may be directly derived from the u-expansion of the former. In particular,

$$\mathcal{C}(u)\hat{\rho}^{\mathsf{M}} = \mathrm{Tr}_{\mathsf{R}}\left(\mathcal{U}(u)\left(\hat{\rho}^{\mathsf{M}}\otimes|0\rangle\langle 0|^{\otimes R}\right)\right) = \sum_{k=0}^{\infty} u^k\mathrm{Tr}_{\mathsf{R}}\left(\mathcal{R}_k\left(\hat{\rho}^{\mathsf{M}}\otimes|0\rangle\langle 0|^{\otimes R}\right)\right). \tag{28}$$

where we have used Supplementary Equation 26. The final expression is exactly the desired form of Supplementary Equation 27, provided we make the identification

$$\mathcal{P}_k\hat{\rho}^{\mathsf{M}} = \mathrm{Tr}_{\mathsf{R}}\left(\mathcal{R}_k\left(\hat{\rho}^{\mathsf{M}}\otimes|0\rangle\langle 0|^{\otimes R}\right)\right). \tag{29}$$

If $k = 0$, then in the main text we have already pointed out that the *null-input superoperator* $\mathcal{P}_0 = \mathcal{C}(0)$ is a CPTP map. Furthermore, notice the expansion identity:

$$\mathcal{C}(u)\hat{\rho}^{\mathsf{M}} = \mathcal{P}_0\hat{\rho}^{\mathsf{M}} + \sum_{k=1}^{\infty} u^k\mathcal{P}_k\hat{\rho}^{\mathsf{M}}, \tag{30}$$

the trace-preserving nature, namely $\mathrm{Tr}(\mathcal{C}(u)\hat{\rho}^{\mathsf{M}}) = \mathrm{Tr}(\mathcal{P}_0\hat{\rho}^{\mathsf{M}}) \equiv 1$, implies the tracelessness of $\mathcal{P}_k$ for all $k \geq 1$, i.e.

$$\mathrm{Tr}(\mathcal{P}_k\hat{\rho}^{\mathsf{M}}) = 0. \tag{31}$$

If we take $\mathcal{P}_k\hat{\rho}^{\mathsf{M}} = \sum_{\alpha=1}^{4^M} c_\alpha^{(k)}\hat{\varrho}_\alpha^{\mathsf{M}}$, where $\hat{\varrho}_\alpha^{\mathsf{M}}$ are the eigenmatrices of superoperators $\mathcal{P}_0\hat{\varrho}_\alpha^{\mathsf{M}} = \lambda_\alpha\hat{\varrho}_\alpha^{\mathsf{M}}$. The decomposition coefficient $c_1^{(k)}$ is the most different one since its associated matrix $\hat{\varrho}_1^{\mathsf{M}} = \hat{\rho}_{\mathrm{FP}}^{\mathsf{M}}$ will remain unchanged when applied by $\mathcal{P}_0$ while other modes decay to zero: $\lim_{n\to\infty}\mathcal{P}_0^n\mathcal{P}_k\hat{\rho}_0^{\mathsf{M}} = c_1^{(k)}\hat{\rho}_{\mathrm{FP}}^{\mathsf{M}}$. As a result,

$$c_1^{(k)} = c_1^{(k)}\mathrm{Tr}(\hat{\rho}_{\mathrm{FP}}^{\mathsf{M}}) = \lim_{n\to\infty}\mathrm{Tr}(\mathcal{P}_0^n\mathcal{P}_k\hat{\rho}_0^{\mathsf{M}}) = \lim_{n\to\infty}\mathrm{Tr}(\mathcal{P}_k\hat{\rho}_0^{\mathsf{M}}) = 0. \tag{32}$$

Therefore, we conclude a very useful property that

$$\mathcal{P}_k\hat{\rho}^{\mathsf{M}} = \sum_{\alpha=2}^{4^M} c_\alpha^{(k)}\hat{\varrho}_\alpha^{\mathsf{M}} \tag{33}$$

for any memory density matrix $\hat{\rho}^{\mathsf{M}}$ and any $k \geq 1$.

2. $\mathcal{R}_k$ and $\mathcal{P}_k$ for linear Hamiltonian encoding scheme by regrouping the BCH formula

We now evaluate the u-expansion of $\mathcal{U}(u)\hat{\rho}^{\mathsf{MR}} = e^{-i\tau\hat{H}(u)}\hat{\rho}^{\mathsf{MR}}e^{i\tau\hat{H}(u)}$. Central to this expansion is the Baker-Campbell-Hausdorff (BCH) formula, which allows us to write this expression in the series form

$$e^{-i\tau\hat{H}(u)}\hat{\rho}^{\mathsf{MR}}e^{i\tau\hat{H}(u)} = \sum_{q=0}^{\infty}\frac{(-i\tau)^q}{q!}[\hat{H}(u),[\cdots[\hat{H}(u),\hat{\rho}^{\mathsf{MR}}]\cdots]] \tag{34}$$

Using the explicit form $\hat{H}(u) = \hat{H}_0 + u\hat{H}_1$, we can compute the superoperator coefficient of any term in the series:

$$\begin{aligned}
\frac{(-i\tau)^1}{1!}:\ [\hat{H}(u),\hat{\rho}^{\mathsf{MR}}] =& [\hat{H}_0,\hat{\rho}^{\mathsf{MR}}] + u^1[\hat{H}_1,\hat{\rho}^{\mathsf{MR}}],\\
\frac{(-i\tau)^2}{2!}:\ [\hat{H}(u),[\hat{H}(u),\hat{\rho}^{\mathsf{MR}}]] =& [\hat{H}_0,[\hat{H}_0,\hat{\rho}^{\mathsf{MR}}]] + u^1\left([\hat{H}_0,[\hat{H}_1,\hat{\rho}^{\mathsf{MR}}]] + [\hat{H}_1,[\hat{H}_0,\hat{\rho}^{\mathsf{MR}}]]\right) + u^2[\hat{H}_1,[\hat{H}_1,\hat{\rho}^{\mathsf{MR}}]],\\
\frac{(-i\tau)^3}{3!}:\ [\hat{H}(u),[\hat{H}(u),[\hat{H}(u),\hat{\rho}^{\mathsf{MR}}]]] =& [\hat{H}_0,[\hat{H}_0,[\hat{H}_0,\hat{\rho}^{\mathsf{MR}}]]]+\\
&+u^1\left([\hat{H}_0,[\hat{H}_0,[\hat{H}_1,\hat{\rho}^{\mathsf{MR}}]]] + [\hat{H}_0,[\hat{H}_1,[\hat{H}_0,\hat{\rho}^{\mathsf{MR}}]]] + [\hat{H}_1,[\hat{H}_0,[\hat{H}_0,\hat{\rho}^{\mathsf{MR}}]]]\right)\\
&+u^2\left([\hat{H}_1,[\hat{H}_1,[\hat{H}_0,\hat{\rho}^{\mathsf{MR}}]]] + [\hat{H}_1,[\hat{H}_0,[\hat{H}_1,\hat{\rho}^{\mathsf{MR}}]]] + [\hat{H}_0,[\hat{H}_1,[\hat{H}_1,\hat{\rho}^{\mathsf{MR}}]]]\right)\\
&+u^3\left([\hat{H}_1,[\hat{H}_1,[\hat{H}_1,\hat{\rho}^{\mathsf{MR}}]]]\right),\\
&\vdots
\end{aligned}$$

Note that each term in the series can be viewed as a series in u^k instead. Furthermore, each appearance of $u\hat{H}_1$ in $\hat{H}(u)$ contributes exactly one factor of u. This allows us to determine the coefficient of u^k in the qth term:

$$u^k \times \frac{(-i\tau)^q}{q!}\sum_{\{\hat{C}_1,\hat{C}_2,\cdots,\hat{C}_q\}}[\hat{C}_1,[\hat{C}_2,[\cdots,[\hat{C}_q,\hat{\rho}^{\mathsf{MR}}]\cdots]]] \tag{35}$$

Here, the summation is over all $\begin{pmatrix} q \\ k \end{pmatrix}$ possible combinations $\{\hat{C}_1,\hat{C}_2,\cdots,\hat{C}_q\}$ which is an ordered set with k instances of $\hat{H}_0$ and $(q-k)$ instances of $\hat{H}_1$. This expression allows us to regroup the BCH formula not by the parameter q as in Supplementary Equation 34, but by powers u^k of the input. We therefore arrive at the desired form of Supplementary Equation 26,

$$\mathcal{U}(u)\hat{\rho}^{\mathsf{MR}} = \sum_{k=0}^{\infty}u^k\mathcal{R}_k\hat{\rho}^{\mathsf{MR}}, \tag{36}$$

with

$$\mathcal{R}_k\hat{\rho}^{\mathsf{MR}} = \sum_{q=k}^{\infty}\frac{(-i\tau)^q}{q!}\sum_{\{\hat{C}_1,\hat{C}_2,\cdots,\hat{C}_q\}}[\hat{C}_1,[\hat{C}_2,[\cdots,[\hat{C}_q,\hat{\rho}^{\mathsf{MR}}]\cdots]]]. \tag{37}$$

3. Functional I/O map: time-invariance and Volterra kernels

Our work in the previous subsection allows us to express the action of individual superoperators $\mathcal{U}(u)$ and $\mathcal{C}(u)$ on a general $\hat{\rho}^{\mathsf{MR}}$ as a u-expansion at every time step. The dynamical map defined by our time-dependent NISQRC framework involves the repeated application of these superoperators for distinct inputs u_n, so that the output at time step n may have a complicated dependence on prior inputs $u_{\leq n}$. We are now in a position to extract this dependence explicitly. To do so, we simply substitute our u-expansions for the superoperators $\mathcal{U}(u)$ and $\mathcal{C}(u)$ into the evolution equation Supplementary Equation 25 defining $\hat{\rho}_n^{\mathsf{MR}}$ at an arbitrary time step n, i.e. $\hat{\rho}_n^{\mathsf{MR}} = \mathcal{U}(u_n)\left(\left(\mathcal{C}(u_{n-1})\cdots\mathcal{C}(u_1)\hat{\rho}_0^{\mathsf{M}}\right)\otimes|0\rangle\langle 0|^{\otimes R}\right)$. Then, the density matrices at time step n attain the formal expression:

$$\hat{\rho}_n^{\mathsf{MR}} = \sum_{k_1,\cdots,k_n=0}^{\infty} u_1^{k_1}\cdots u_{n-1}^{k_{n-1}}u_n^{k_n}\times\mathcal{R}_{k_n}\left(\left(\mathcal{P}_{k_{n-1}}\cdots\mathcal{P}_{k_1}\hat{\rho}_0^{\mathsf{M}}\right)\otimes|0\rangle\langle 0|^{\otimes R}\right) \tag{38}$$

Before evaluating the readout features $x_j(n)$, we need to simplify Supplementary Equation 38 as much as possible. The starting point is first looking at the simplest contribution from term u_{n-1} to $\hat{\rho}_n^{\mathsf{MR}}$ (namely the one-step backwards linear contribution). This means that we can let $k_1 = \cdots = k_{n-2} = k_n = 0$ and $k_{n-1} = 1$. The associated prefactor is

$$\mathcal{R}_0\left(\left(\mathcal{P}_1\mathcal{P}_0^{n-2}\hat{\rho}_0^{\mathsf{M}}\right)\otimes|0\rangle\langle 0|^{\otimes R}\right), \tag{39}$$

Similarly, analyzing contribution from term u_n to $\hat{\rho}_{n+1}^{\mathsf{MR}}$ (that is, let $k_1 = \cdots = k_{n-1} = k_{n+1} = 0$ and $k_n = 1$) gives associated prefactor

$$\mathcal{R}_0\left(\left(\mathcal{P}_1\mathcal{P}_0^{n-1}\hat{\rho}_0^{\mathsf{M}}\right)\otimes|0\rangle\langle 0|^{\otimes R}\right). \tag{40}$$

In principle, $\mathcal{P}_0^{n-2}\hat{\rho}_0^{\mathsf{M}} \neq \mathcal{P}_0^{n-1}\hat{\rho}_0^{\mathsf{M}}$ and therefore term Supplementary Equation 39 and Supplementary Equation 40 are analytically different. However, with the existence of fixed point state

$$\lim_{n\to\infty}\mathcal{P}_0^n\hat{\rho}_0^{\mathsf{M}} = \hat{\rho}_{\mathrm{FP}}^{\mathsf{M}}, \tag{41}$$

it ensures the approximation

$$\mathcal{P}_0^{n-2}\hat{\rho}_0^{\mathsf{M}} \approx \hat{\rho}_{\mathrm{FP}}^{\mathsf{M}} \approx \mathcal{P}_0^{n-1}\hat{\rho}_0^{\mathsf{M}}, \tag{42}$$

and hence Supplementary Equation 39 and Supplementary Equation 40 are asymptotically the same. Such property is usually referred as (asymptotic) *time-invariance*. In fact, we can further weaken this requirement that all peripheral spectrum λ_α (namely those eigenvalue with magnitude $|\lambda_\alpha| = 1$) are $\lambda_\alpha = 1$. For example, for a fully connected quantum reservoir with $M+R$ qubits, if $J_{i,i'}$ are constant for every coupling pair and η_i^x, η_i^z are also constant for every qubit, then the numerical results show that the fixed points of $\hat{\rho}_0^{\mathsf{M}}$ will have a degeneracy of *Catalan numbers* $\frac{(2M)!}{M!(M+1)!}$. In this case, the fixed point $\lim_{n\to\infty}\mathcal{P}_0^n\hat{\rho}_0^{\mathsf{M}} = \hat{\rho}_{\mathrm{FP}}^{\mathsf{M}}$ still exists but will depend on initial state $\hat{\rho}_0^{\mathsf{M}}$.

The above calculation works for any contribution terms in $\hat{\rho}_n^{\mathsf{MR}}$. This establishes all analytical expressions of Volterra series kernels. The leading order kernels can be written down compactly:

- The zero-th order Volterra kernel:

$$h_0^{(j)} = \mathrm{Tr}\left(\hat{M}_j\mathcal{R}_0\left(\hat{\rho}_{\mathrm{FP}}^{\mathsf{M}}\otimes|0\rangle\langle 0|^{\otimes R}\right)\right), \tag{43}$$

- The first order Volterra kernel ($n_1 \geq 0$):

$$h_1^{(j)}(n_1) = \begin{cases} \mathrm{Tr}\left(\hat{M}_j\mathcal{R}_1\left(\hat{\rho}_{\mathrm{FP}}^{\mathsf{M}}\otimes|0\rangle\langle 0|^{\otimes R}\right)\right), & \text{if } n_1 = 0, \\ \mathrm{Tr}\left(\hat{M}_j\mathcal{R}_0\left(\left(\mathcal{P}_0^{n_1-1}\mathcal{P}_1\hat{\rho}_{\mathrm{FP}}^{\mathsf{M}}\right)\otimes|0\rangle\langle 0|^{\otimes R}\right)\right), & \text{if } n_1 \neq 0, \end{cases} \tag{44}$$

- And the second order Volterra kernel ($n_2 \geq n_1 \geq 0$):

$$h_2^{(j)}(n_1, n_2) = \begin{cases} \mathrm{Tr}\Big(\hat{M}_j \mathcal{R}_2\Big(\hat{\rho}^{\mathsf{M}}_{\mathrm{FP}} \otimes |0\rangle\langle 0|^{\otimes R}\Big)\Big), & \text{if } n_1 = 0, n_2 = 0, \\ \mathrm{Tr}\Big(\hat{M}_j \mathcal{R}_1\Big((\mathcal{P}_0^{n_2-1}\mathcal{P}_1\hat{\rho}^{\mathsf{M}}_{\mathrm{FP}}) \otimes |0\rangle\langle 0|^{\otimes R}\Big)\Big), & \text{if } n_1 = 0, n_2 > 0, \\ \mathrm{Tr}\Big(\hat{M}_j \mathcal{R}_0\Big((\mathcal{P}_0^{n_2-1}\mathcal{P}_2\hat{\rho}^{\mathsf{M}}_{\mathrm{FP}}) \otimes |0\rangle\langle 0|^{\otimes R}\Big)\Big), & \text{if } n_1 = n_2 > 0, \\ \mathrm{Tr}\Big(\hat{M}_j \mathcal{R}_0\Big((\mathcal{P}_0^{n_1-1}\mathcal{P}_1\mathcal{P}_0^{n_2-n_1-1}\mathcal{P}_1\hat{\rho}^{\mathsf{M}}_{\mathrm{FP}}) \otimes |0\rangle\langle 0|^{\otimes R}\Big)\Big), & \text{if } 0 < n_1 < n_2. \end{cases} \tag{45}$$

These kernel expressions show that if the reservoir output nontrivially depends on the history, then $\mathcal{P}_k\hat{\rho}^{\mathsf{M}}_{\mathrm{FP}} \neq 0$ for some $k \geq 1$. Equivalently, if $\mathcal{P}_k\hat{\rho}^{\mathsf{M}}_{\mathrm{FP}} = 0$ for all $k \geq 1$, then $h_k^{(j)}(n_1, n_2, \cdots, n_k) \neq 0$ only if $n_1 = n_2 = \cdots = n_k = 0$.

We emphasize that even though $h_k^{(j)}(n_1, n_2, \cdots, n_k)$ (e.g., Supplementary Equation 43-45 are kernels of real values $x_j(n)$, these kernels all take the form of $\mathrm{Tr}(\hat{M}_j \cdot)$, where "$\cdot$" are always quantum operators which expand $\hat{\rho}^{\mathsf{MR}}_n$. Therefore, it is intuitive to write these quantum operators into

$$\hat{\rho}^{\mathsf{MR}}_n = \sum_{k=0}^{\infty}\sum_{n_1=0}^{\infty}\cdots\sum_{n_k=n_{k-1}}^{\infty} \hat{h}_k(n_1, \cdots, n_k)\prod_{\kappa=1}^{k} u_{n-n_\kappa}. \tag{46}$$

Those quantum operators $\hat{h}_k$ are the central objects in the u-expansion and all classical kernels in Eq. (1) are $h_k^{(j)} = \mathrm{Tr}(\hat{M}_j\hat{h}_k)$, justifying the nomenclature of Quantum Volterra Theory used for the entire framework in the main text.

We note that as we proved in Supplementary Equation 17, $\lim_{n\to\infty}\hat{\rho}^{\mathsf{MR}}_n = \frac{\hat{I}^{\otimes L}}{2^L}$. This can also be understood through the Volterra expansion. Recall Supplementary Equation 13, i.e. $\hat{\rho}^{\mathsf{MR}}_n = \mathcal{U}(u_n)\mathcal{M}\cdots\mathcal{U}(u_2)\mathcal{M}\mathcal{U}(u_1)\rho^{\mathsf{MR}}_0$. By plugging Supplementary Equation 26 and Supplementary Equation 27, we get

$$\hat{\rho}^{\mathsf{MR}}_n = \sum_{k_1,k_2,\cdots,k_n=0}^{\infty} u_1^{k_1}u_2^{k_2}\cdots u_n^{k_n}\mathcal{R}_{k_n}\mathcal{M}\mathcal{R}_{k_{n-1}}\cdots\mathcal{M}\mathcal{R}_{k_2}\mathcal{M}\mathcal{R}_{k_1}\hat{\rho}^{\mathsf{MR}}_0 \tag{47}$$

All $\mathcal{P}_k\hat{\rho}^{\mathsf{M}}$ in previous Volterra analysis must be replaced with $\mathcal{M}\mathcal{R}_k\hat{\rho}^{\mathsf{MR}}$. However, $\mathcal{M}\mathcal{R}_0\hat{\rho}^{\mathsf{MR}}_{\mathrm{FP}} = \hat{\rho}^{\mathsf{MR}}_{\mathrm{FP}}$ implies $\hat{\rho}^{\mathsf{MR}}_{\mathrm{FP}} = \frac{\hat{I}^{\otimes L}}{2^L}$, and thus all Volterra kernels must vanish, since the identity makes all commutator terms in Supplementary Equation 37 vanish exactly. This reproduces the null response of a NISQRC architecture in the absence of the reset operation.

4. u-expansion and Volterra kernels for dissipative quantum systems

Thus far, we have demonstrated how the u-expansion can be performed for a CPTP map without explicit dissipative evolution. In this subsection, we extend this analysis to account for dissipative quantum systems, as is relevant for practical NISQRC implementations.

In particular, we wish to now consider the evolution governed by the general CPTP map $e^{\tau\mathcal{L}(u)}\hat{\rho}^{\mathsf{MR}}$, where $\mathcal{L}$ is the Liouvillian superoperator, for example of the type introduced in Eq. (6) of the main text. We first note that the BCH formula of Supplementary Equation 34 can be rewritten compactly in the dissipation free case by first introducing the *adjoint action* $[\hat{X}, \hat{Y}] = \mathrm{ad}_{\hat{X}}\hat{Y}$ for arbitrary matrices $\hat{X}, \hat{Y}$. With this notation, the BCH formula becomes:

$$e^{-i\tau\hat{H}(u)}\hat{\rho}e^{i\tau\hat{H}(u)} = e^{-i\tau[\hat{H}(u),\cdot]}\hat{\rho} = \sum_{q=0}^{\infty}\frac{(-i\tau)^q}{q!}\mathrm{ad}^q_{\hat{H}(u)}\hat{\rho}. \tag{48}$$

In presence of dissipation, the adjoint action allows us to write the operation of the Liouvillian $\mathcal{L}(u)$, $e^{\tau\mathcal{L}(u)}\hat{\rho}^{\mathsf{MR}}$, in the form:

$$\begin{aligned} e^{\tau\mathcal{L}(u)}\hat{\rho}^{\mathsf{MR}} =& e^{-i\tau\left(\left(\mathrm{ad}_{\hat{H}_0}+i\mathcal{D}_{\mathrm{T}}\right)+u\,\mathrm{ad}_{\hat{H}_1}\right)}\hat{\rho}^{\mathsf{MR}} \\ =& \hat{\rho}^{\mathsf{MR}} + \frac{(-i\tau)^1}{1!}\left(\left(\mathrm{ad}_{\hat{H}_0}+i\mathcal{D}_{\mathrm{T}}\right)+u\,\mathrm{ad}_{\hat{H}_1}\right)\hat{\rho}^{\mathsf{MR}} + \frac{(-i\tau)^2}{2!}\left(\left(\mathrm{ad}_{\hat{H}_0}+i\mathcal{D}_{\mathrm{T}}\right)+u\,\mathrm{ad}_{\hat{H}_1}\right)^2\hat{\rho}^{\mathsf{MR}} + \cdots. \end{aligned} \tag{49}$$

where we have also used the explicit form of $\hat{H}(u) = \hat{H}_0 + u\hat{H}_1$, and where $\mathcal{D}_{\mathrm{T}}$ describes T_1 decay of all qubits in the QRC with a rate γ, see Eq. (6) of the main text.

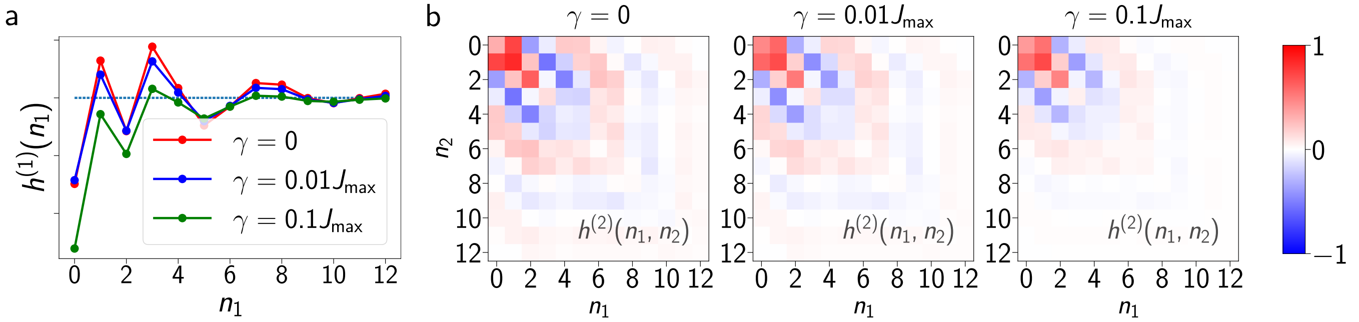

Supplementary Figure 3. The first and second order Volterra Kernel example in a $(2+1)$-qubit quantum reservoir with fundamental decay γ. The parameters are chosen to be constant $J_{i,i'} = \eta_i^x = \eta_i^z = 1.3$ (in unit $1/\tau$) for simplicity. (The same as Fig. 2 of the main text). (a) The first order kernel $h_1^{(1)}(n_1)$, with decay rate $\gamma = 0$ (no decay, in red), $0.01J_{\max}$ (in blue), $0.1J_{\max}$ (in green). (b) The second order kernel $h_2^{(1)}(n_1, n_1)$, with decay rate $\gamma = 0$ (no decay, left), $0.01J_{\max}$ (middle), $0.1J_{\max}$ (right). The first and second order kernel without decay is exactly the kernel in Fig. 2(a) of the main text.

Based on this formalism, we are now able to read off the u-expansion for the CPTP map $e^{\tau\mathcal{L}(u)}\hat{\rho}^{\mathsf{MR}}$ by regrouping modified BCH formula:

$$e^{\tau\mathcal{L}(u)}\hat{\rho}^{\mathsf{MR}} = \sum_{k=0}^{\infty} u^k \mathcal{Q}_k \hat{\rho}^{\mathsf{MR}}, \tag{50}$$

where the superoperators are defined as

$$\begin{aligned}
\mathcal{Q}_0\hat{\rho}^{\mathsf{MR}} &= \hat{\rho}^{\mathsf{MR}} - i\tau\left(\mathrm{ad}_{\hat{H}_0} + i\mathcal{D}_{\mathrm{T}}\right)\hat{\rho}^{\mathsf{MR}} - \frac{\tau^2}{2!}\left(\mathrm{ad}_{\hat{H}_0} + i\mathcal{D}_{\mathrm{T}}\right)^2\hat{\rho}^{\mathsf{MR}} + \cdots \\
&= \hat{\rho}^{\mathsf{MR}} - i\tau\left([\hat{H}_0, \hat{\rho}^{\mathsf{MR}}] + i\mathcal{D}_{\mathrm{T}}\hat{\rho}^{\mathsf{MR}}\right) - \frac{\tau^2}{2!}\left([\hat{H}_0, [\hat{H}_0, \hat{\rho}^{\mathsf{MR}}]] + i[\hat{H}_0, \mathcal{D}_{\mathrm{T}}\hat{\rho}^{\mathsf{MR}}] + i\mathcal{D}_{\mathrm{T}}[\hat{H}_0, \hat{\rho}^{\mathsf{MR}}] - \mathcal{D}_{\mathrm{T}}^2\hat{\rho}^{\mathsf{MR}}\right) + \cdots \\
&\equiv e^{\tau\mathcal{L}(0)}\hat{\rho}^{\mathsf{MR}}, \\
\mathcal{Q}_1\hat{\rho}^{\mathsf{MR}} &= -i\tau\,\mathrm{ad}_{\hat{H}_1}\hat{\rho}^{\mathsf{MR}} - \frac{\tau^2}{2!}\left(\left(\mathrm{ad}_{\hat{H}_0} + i\mathcal{D}_{\mathrm{T}}\right)\mathrm{ad}_{\hat{H}_1} + \mathrm{ad}_{\hat{H}_1}\left(\mathrm{ad}_{\hat{H}_0} + i\mathcal{D}_{\mathrm{T}}\right)\right)\hat{\rho}^{\mathsf{MR}} + \cdots, \\
&= -i\tau[\hat{H}_1, \hat{\rho}^{\mathsf{MR}}] - \frac{\tau^2}{2!}\left([\hat{H}_0, [\hat{H}_1, \hat{\rho}^{\mathsf{MR}}]] + [\hat{H}_1, [\hat{H}_0, \hat{\rho}^{\mathsf{MR}}]] + i\mathcal{D}_{\mathrm{T}}[\hat{H}_1, \hat{\rho}^{\mathsf{MR}}] + i[\hat{H}_1, \mathcal{D}_{\mathrm{T}}\hat{\rho}^{\mathsf{MR}}]\right) + \cdots, \\
\mathcal{Q}_2\hat{\rho}^{\mathsf{MR}} &= -\frac{\tau^2}{2!}\mathrm{ad}_{\hat{H}_1}^2\hat{\rho}^{\mathsf{MR}} + \cdots = -\frac{\tau^2}{2!}[\hat{H}_1, [\hat{H}_1, \hat{\rho}^{\mathsf{MR}}]] + \cdots. \\
&\vdots
\end{aligned}$$

The knowledge of superoperators $\{\mathcal{Q}_k\}$ therefore allows us to compute the Volterra kernels for NISQRC in the presence of dissipation. Some numerical simulations of the first- and second-order kernels are shown in Supplementary Figure 3, with increasing decay rate γ (for other QRC parameters, see caption). We see that dissipation can reduce the amplitude of the QRC response to the input - governed by the amplitude of the kernels - in particular to past inputs indicated by increasing values of n_1, n_2. Hence dissipation can reduce the memory of the NISQRC framework. However, even for modest amounts of dissipation smaller than the strength of Hamiltonian terms, the kernels are certainly far from trivial, retaining their qualitative features with a non-zero memory term. This indicates the applicability of the NISQRC framework to contemporary dissipative quantum systems used as QRCs.

Supplementary Note 4: Fading memory modes

For any $k \geq 1$, we define for each $\alpha' \in \mathbb{N}$

$$\mathcal{P}_k\hat{\varrho}_{\alpha'}^{\mathsf{M}} = \sum_{\alpha=2}^{4^M} c_{\alpha\alpha'}^{(k)}\hat{\varrho}_{\alpha}^{\mathsf{M}}. \tag{51}$$

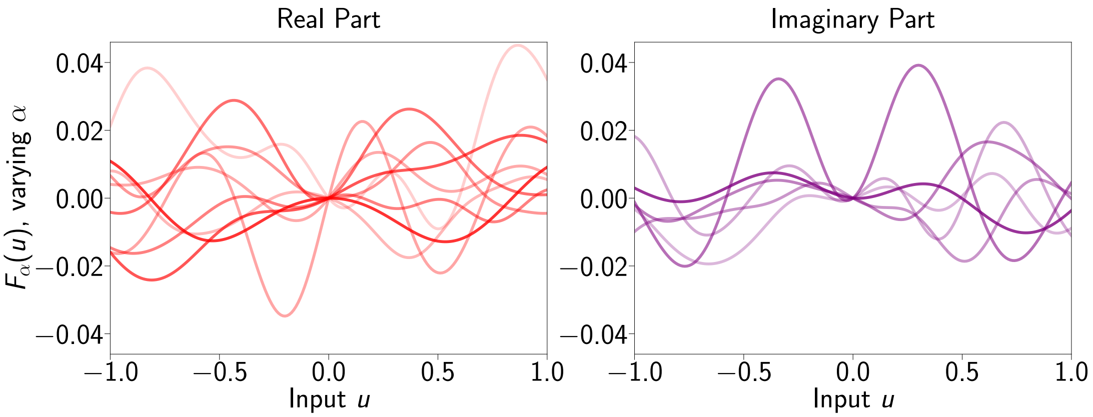

Supplementary Figure 4. $4^M-1=15$ internal features $F_\alpha(u)$ in a $(2+1)$-qubit QRC. The hyperparameters are $(J_{\max};\eta^x,\varepsilon^x_{\rm rms};\eta^z,\varepsilon^z_{\rm rms})=(1;3,1;4,2)$ in unit $1/\tau$. $F_\alpha(u)$ is potentially a complex-valued function. The eigenvalues of $\mathcal{P}_0$ appears in pair: λ_α being an eigenvalue implies λ_α^* also being an eigenvalue. Therefore, both function $F_\alpha(u)$ and its conjugate $F_\alpha(u)^*$ are internal features. So the of 4^M-1 internal feature functions contains exactly 4^M-1 independent real-function. They are plotted separately in sense of of real part (red solid lines) and imaginary part (purple solid lines). The darker the line is, the larger the corresponding eigenvalue norm $|\lambda_\alpha|$ is and the slower this internal feature fades.

Notice that $c^{(k)}_{1\alpha'}\equiv 0$ for any $k\geq 1$ due to the tracelessness of $\mathcal{P}_k$ (recall $\hat{\varrho}^{\rm M}_1=\hat{\rho}^{\rm M}_{\rm FP}$ by definition), thus the summation begins with $\alpha=2$. Contributions from $(u_{n-n_1},u_{n-n_2},\cdots,u_{n-n_P})$, where $0<n_1<n_2<\cdots<n_P$, is given by

$$
\begin{aligned}
&\sum_{k_1,\cdots,k_P=1}^{\infty} h^{(j)}_{k_1+\cdots+k_P}(n_1^{\otimes k_1},\cdots,n_P^{\otimes k_P})\times u^{k_1}_{n-n_1}\cdots u^{k_P}_{n-n_P}\\
=&\sum_{k_1,\cdots,k_P=1}^{\infty} \mathrm{Tr}\Big(\hat{M}_j\mathcal{R}_0\Big(\mathcal{P}_0^{n_1-1}\mathcal{P}_{k_1}\cdots\mathcal{P}_0^{n_P-n_{P-1}-1}\mathcal{P}_{k_P}\hat{\rho}^{\rm M}_{\rm FP}\otimes|0\rangle\langle 0|^{\otimes R}\Big)\Big)\times u^{k_1}_{n-n_1}\cdots u^{k_P}_{n-n_P}\\
=&\sum_{k_1,\cdots,k_P=1}^{\infty} \mathrm{Tr}\left(\hat{M}_j\mathcal{R}_0\left(\mathcal{P}_0^{n_1-1}\mathcal{P}_{k_1}\cdots\mathcal{P}_0^{n_P-n_{P-1}-1}\left(\sum_{\alpha_P=2}^{4^M}c^{(k_P)}_{\alpha_P 1}\hat{\varrho}^{\rm M}_{\alpha_P}\right)\otimes|0\rangle\langle 0|^{\otimes R}\right)\right)\times u^{k_1}_{n-n_1}\cdots u^{k_P}_{n-n_P}\\
&\vdots\\
=&\sum_{k_1,\cdots,k_P=1}^{\infty}\sum_{\alpha_1,\cdots,\alpha_P=2}^{4^M} \mathrm{Tr}\Big(\hat{M}_j\mathcal{R}_0\Big(\lambda_{\alpha_1}^{n_1-1}c^{(k_1)}_{\alpha_1\alpha_2}\cdots\lambda_{\alpha_P}^{n_P-n_{P-1}-1}c^{(k_P)}_{\alpha_P 1}\hat{\varrho}^{\rm M}_{\alpha_1}\otimes|0\rangle\langle 0|^{\otimes R}\Big)\Big)\times u^{k_1}_{n-n_1}\cdots u^{k_P}_{n-n_P}\\
=&\sum_{\alpha_1,\cdots,\alpha_P=2}^{4^M}\lambda_{\alpha_1}^{n_1-1}\cdots\lambda_{\alpha_P}^{n_P-1}\sum_{k_1,\cdots,k_P=1}^{\infty}c^{(k_1)}_{\alpha_1\alpha_2}\cdots c^{(k_P)}_{\alpha_P 1}\mathrm{Tr}\Big(\hat{M}_j\mathcal{R}_0\Big(\hat{\varrho}^{\rm M}_{\alpha_1}\otimes|0\rangle\langle 0|^{\otimes v}\Big)\Big)\times u^{k_1}_{n-n_1}\cdots u^{k_P}_{n-n_P}.
\end{aligned}
\tag{52}
$$

Namely, we can decompose the contributions from $(u_{n-n_1},u_{n-n_2},\cdots,u_{n-n_P})$ to $x_j(n)$ into $(4^M-1)^P$ memory modes of internal features:

$$
\begin{aligned}
x_j(n)=&\sum_{\alpha_1,\alpha_2,\cdots,\alpha_P=2}^{4^M}\nu^{(j)}_{\alpha_1}\lambda_{\alpha_1}^{n_1-1}\lambda_{\alpha_2}^{n_2-n_1-1}\cdots\lambda_{\alpha_P}^{n_P-n_{P-1}-1}\\
&\times F_{\alpha_1,\alpha_2,\cdots,\alpha_P}(u_{n-n_1},u_{n-n_2},\cdots,u_{n-n_P})+\cdots
\end{aligned}
\tag{53}
$$

where the cross-step internal features

$$
F_{\alpha_1,\alpha_2,\cdots,\alpha_P}(u_{n-n_1},u_{n-n_2},\cdots,u_{n-n_P})=\sum_{k_1,k_2\cdots,k_P=1}^{\infty}c^{(k_1)}_{\alpha_1\alpha_2}c^{(k_2)}_{\alpha_2\alpha_3}\cdots c^{(k_P)}_{\alpha_P 1}u^{k_1}_{n-n_1}u^{k_2}_{n-n_2}\cdots u^{k_P}_{n-n_P}.
\tag{54}
$$

Thanks to the *fading memory* property, namely that λ_α^n converges to zero if $\alpha\geq 2$, the more history steps one monomial term in Volrerra series Eq. (1) involves, the less it contributes to the current-time readout features $x_j(n)$. Therefore, it will be

illustrative for this Supplementary Note to mostly be concerned with a single past time step's contribution. To be more specific, if we focus on the contribution from u_{n-p} to $x_j(n)$ (where $p \geq 1$). For this history record contribution,

$$\sum_{k=1}^{\infty} h_k^{(j)}(p^{\otimes k})\, u_{n-p}^k = \sum_{\alpha=2}^{4^M} \nu_\alpha^{(j)} \lambda_\alpha^{p-1} F_\alpha(u_{n-p}). \tag{55}$$

Each coefficient $\nu_\alpha^{(j)} = \mathrm{Tr}\left(\hat{M}_j \mathcal{R}_0\left(\hat{\varrho}_\alpha^{\mathsf{M}} \otimes |0\rangle\langle 0|^{\otimes R}\right)\right)$ characterizes a different observable $\hat{M}_j$'s response to different *internal features* $F_\alpha(u)$ where

$$F_\alpha(u) = \sum_{k=1}^{\infty} c_{\alpha 1}^{(k)} u^k. \tag{56}$$

Especially, if $\alpha = 1$, then $c_{\alpha 1}^{(k)} = 0$ for any $k \geq 1$, according to Supplementary Equation 32. That is why the summation over α starts from $\alpha = 2$, and it only gives us $4^M - 1$ internal features (see Supplementary Figure 4 as an example).

Supplementary Note 5: Relation between functional-independence and Jacobian rank

In this Supplementary Note we analyze the functional-independence of readout features in the NISQRC framework. Assuming a finite-dimensional input space $\mathbf{u} = (u_1, u_2, \cdots, u_N)$, then NISQRC readout features define K finite-dimensional functions (assuming $K \leq N$), $x_k(u_1, u_2, \cdots, u_N)$, $k \in \{0, \cdots, K-1\}$. An important question is whether these K functions are in fact functionally-independent from one another, since their inter-dependence can impose a limitation on their usefulness for functional approximation using the NISQRC framework.

If the K functions are functionally-dependent, namely there exists some K-variate function G such that:

$$G(x_0(\mathbf{u}), x_1(\mathbf{u}), \cdots, x_{K-1}(\mathbf{u})) \equiv 0. \tag{57}$$

Take gradients

$$\begin{pmatrix} \frac{\partial G}{\partial u_1}(\mathbf{u}) \\ \frac{\partial G}{\partial u_2}(\mathbf{u}) \\ \vdots \\ \frac{\partial G}{\partial u_N}(\mathbf{u}) \end{pmatrix} = \begin{pmatrix} \frac{\partial x_0}{\partial u_1}(\mathbf{u}) & \frac{\partial x_1}{\partial u_1}(\mathbf{u}) & \cdots & \frac{\partial x_{K-1}}{\partial u_1}(\mathbf{u}) \\ \frac{\partial x_0}{\partial u_2}(\mathbf{u}) & \frac{\partial x_1}{\partial u_2}(\mathbf{u}) & \cdots & \frac{\partial x_{K-1}}{\partial u_2}(\mathbf{u}) \\ \vdots & \vdots & \ddots & \vdots \\ \frac{\partial x_0}{\partial u_N}(\mathbf{u}) & \frac{\partial x_1}{\partial u_N}(\mathbf{u}) & \cdots & \frac{\partial x_{K-1}}{\partial u_N}(\mathbf{u}) \end{pmatrix} \begin{pmatrix} \frac{\partial G}{\partial x_0}(x_0, x_1, \cdots, x_{K-1}) \\ \frac{\partial G}{\partial x_1}(x_0, x_1, \cdots, x_{K-1}) \\ \vdots \\ \frac{\partial G}{\partial x_{K-1}}(x_0, x_1, \cdots, x_{K-1}) \end{pmatrix} = 0, \tag{58}$$

then gradients $\nabla_{\mathbf{u}} x_0(\mathbf{u})$, $\nabla_{\mathbf{u}} x_1(\mathbf{u})$, $\cdots$, $\nabla_{\mathbf{u}} x_{K-1}(\mathbf{u})$ must be linearly dependent at all points. Therefore, if $\{x_j(\mathbf{u})\}_{j\in[K]}$ are functionally-dependent, then the gradients $\{\nabla_{\mathbf{u}} x_j(\mathbf{u})\}_{j\in[K]}$ must be linearly-dependent. Equivalently, it suffices to prove the functional-independence of $\{x_j(\mathbf{u})\}_{j\in[K]}$ by showing that $\{\nabla_{\mathbf{u}} x_j(\mathbf{u})\}_{j\in[K]}$ are linearly-independent at almost all points $\mathbf{u}$.

Now we argue by contradiction that $K-1$ gradients of readout features $x_j(n) = \mathrm{Tr}\left(\hat{M}_j \hat{\rho}_n^{\mathsf{MR}}\right)$ are functionally-independent if there is no particular symmetry in the reservoir. We first select $\{\hat{M}_j\}$ as the moment representation to remove the trivial functional dependence that their summation is constant. Suppose there exists coefficients $c_1, c_2, \cdots, c_{K-1}$ such that $\sum_{j=1}^{K-1} c_j \nabla \mathcal{F}_j(u_{\leq n}) = 0$. Notice that

$$\frac{\partial x_j}{\partial u_{n-p}} = \mathrm{Tr}\left(\hat{M}_j \frac{\partial \hat{\rho}_n^{\mathsf{MR}}}{\partial u_{n-p}}\right), \tag{59}$$

then $\sum_{j=1}^{K-1} c_j \nabla \mathcal{F}_j(u_{\leq n}) = 0$ implies that

$$\sum_{j=1}^{K-1} c_j \mathrm{Tr}\left(\hat{M}_j \frac{\partial \hat{\rho}_n^{\mathsf{MR}}}{\partial u_{n-p}}\right) = \mathrm{Tr}\left(\left(\sum_{j=1}^{K-1} c_j \hat{M}_j\right) \frac{\partial \hat{\rho}_n^{\mathsf{MR}}}{\partial u_{n-p}}\right) \equiv 0, \tag{60}$$

for all non-negative integer $p \in \mathbb{N}$. For generic input sequence $\{u_{-\infty}, \cdots, u_{n-1}, u_n\}$, there doesn't exists such observable $\sum_{j=1}^{K-1} c_j \hat{M}_j$ such that expectations of $\frac{\partial \hat{\rho}_n^{\mathsf{MR}}}{\partial u_{n-p}}$ for any $p \in \mathbb{N}$ always vanish, which is a contradiction.

This results shows that in principle, the linear combination of quantum probability readout will yield a function family whose gradient space is much more abundant, because usually the feature number $K-1$ is much larger than the readout qubit number R.

Supplementary Note 6: Channel equalization: background and training details

In this Supplementary Note, we provide some more details of the channel equalization task used as an example of time-dependent processing.

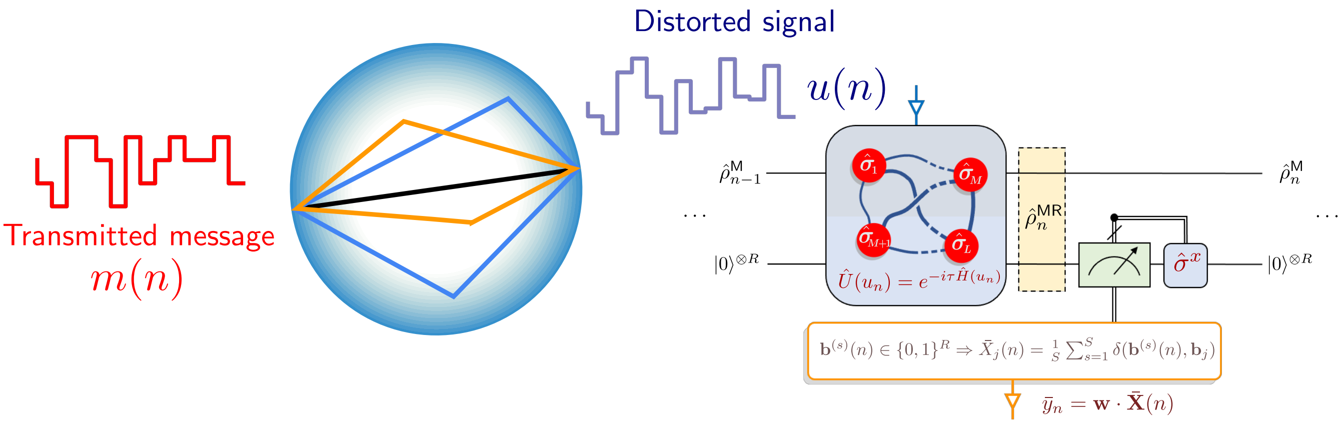

Supplementary Figure 5. Schematic of the goal of the channel equalization task, and a representative implementation using a QRC under the NISQRC framework.

As mentioned in Results' subsection "Practical machine learning using temporal data" of the main text, the channel equalization task requires accurately reconstructing a temporally-varying message $m(n)$ from its corrupted copy $u(n)$ after transmission. For the instance we consider, the distortion of the transmitted signal is modeled via the action of a linear kernel $h(n)$, nonlinear mixing f and additive Gaussian noise ϵ_0:

$$u(n) = f\left(\sum_{n_1=0}^{7} h(n_1)m(n-n_1)\right) + \epsilon_0. \tag{61}$$

We choose a kernel $h \in \mathbb{R}^8$, whose elements we now specify as $h = [1.0, 0.18, -0.1, 0.091, -0.05, 0.04, 0.03,\ 0.01]$. The nonlinear distortion is modeled by the polynomial $f(x) = x + 0.06x^2 - 0.01x^3$, while the additive noise is parameterized as $\epsilon_0 \in \mathcal{N}\left(0, 10^{\frac{-\mathrm{SNR}}{10}}\right)$. The coefficients in h and f are from the channel equalization task presented in Ref. [5]. We remove the leading two coefficients of h which represent dependence on future two steps of message symbols. We also increase the nonlinearity in quadratic part of f to make it non-invertible. Recovering $m(n)$ from $u(n)$ therefore requires a nonzero memory time (to undo the linear kernel), nonlinear processing (to undo the polynomial f), and filtering (to remove added noise). In this simulated scenario where $h(n)$ and $f(x)$ are known, the distortion can be inverted up to the additive noise ϵ_0, thus providing a theoretical bound on the minimum achievable error rate.

We select a $(2+4)$ qubit reservoir, namely with $M = 2$ memory qubits and $R = 4$ readout qubits. The NISQRC Hamiltonian is as given in Eq. (7). We now also detail hyperparameters defining this Hamiltonian for the instance analyzed in Fig. 3 of the main text. In unit of $1/\tau$, the single-qubit terms are defined by hyperparameters $\eta^z = \varepsilon^z_{\mathrm{rms}} = 0.5$ and $\eta^x = \varepsilon^x_{\mathrm{rms}} = 2$. The interaction strengths $J_{i,i'}$ are uniformly sampled from $[0, 1]$, but individual couplings are turned off when analyzing the different QRC connectivities.

Finally, the $R = 4$ readout qubits imply that at each time step n we acquire $K = 2^4 = 16$ readout features $\{\bar{X}_j(n)\}_{j\in[K]}$. A final processing step is the application of a logistic regression layer to these QRC readout features,

$$y_n = \underset{m\in\{-3,-1,1,3\}}{\mathrm{argmax}} \quad \sigma(\mathbf{w}_m \cdot \bar{\mathbf{X}}(n)). \tag{62}$$

for computing and minimizing the cross-entropy loss, where $\mathbf{w} \in \mathbb{R}^{4\times K}$ and $\bar{\mathbf{X}}(n) \in \mathbb{R}^K$. The results of testing using this scheme are depicted in Fig. 3 of the main text.

We compare NISQRC error rates with two meaningful bounds in Fig. 3(a), those derived from the theoretical direct inverse and numerical logistic regression. The lowest possible error rate is that achievable with the direct inverse, which assumes one knows the distorting channel exactly: $y_{\mathrm{DI},n} = \sum_{n_1} h^{-1}(n_1) f^{-1}(u(n-n_1))$, where h^{-1} is the inverse of linear transformation h. The noise term ϵ_0 in Supplementary Equation 61 leads to a non-zero error rate for direct inverse.

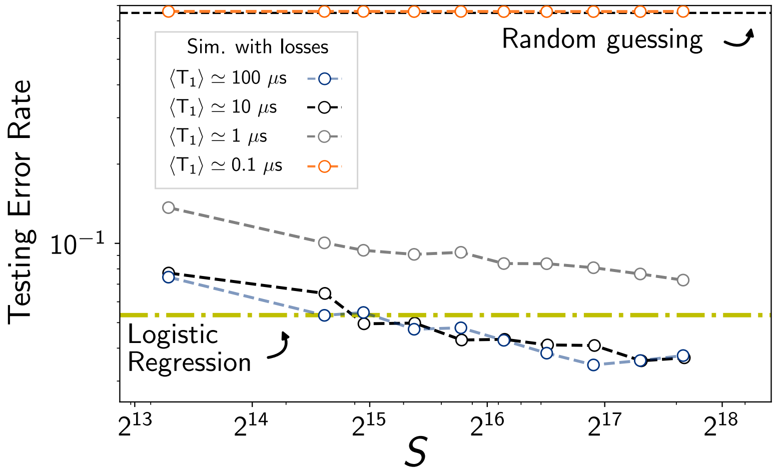

Supplementary Figure 6. Testing error rates for the CE task in the main text, Fig. 4 as a function of number of shots S using simulations of the *ibm_algiers*, now for varying qubit coherence times. Details on coherence time values are provided in the text. For comparison, we plot the testing error rate of logistic regression (yellow line), as well as random guessing (black dashed line).

As an upper bound we consider classical logistic regression applied to the current input value $u(n)$, equivalent to a one-layer perceptron with a softmax activation function. Specifically, we the output is $y_{\mathrm{LR},n} = \mathrm{argmax}_m\, \sigma(\mathbf{w}_{\mathrm{LR},m} \cdot \mathbf{u}(n))$, where weights $\{\mathbf{w}_{\mathrm{LR},m}\}_{m\in\{\pm1,\pm3\}}$ are trained by minimizing the cross-entropy loss over the same training set used for NISQRC. Since this results in a linear and memory-less map, improvements in error-rate beyond this upper-bound indicate useful processing done through NISQRC.

Supplementary Note 7: IBM Device simulations as a function of qubit coherence times

In this Supplementary Note we provide supplementary simulation results for the IBM device analyzed in the main text. From Fig. 4(b) in the main text, we found that actual device results matched ideal results (in the absence of any losses) very well. Since we are primarily interested in the role of finite qubit coherence times, we now consider the role of a loss model that accounts for finite qubit T_1 and T_2 times. In particular, we consider normal distributions $T_1 \in \mathcal{N}(\langle T_1\rangle, \sigma_{T_1})$. and $T_2 \in \mathcal{N}(\langle T_2\rangle, \sigma_{T_2})$ for the $L = 7$ qubit chain. We start with an initial distribution of coherence times consistent with the actual *ibm_algiers* device from which experimental results are shown in the main text; here $\langle T_1\rangle \simeq 100\ \mu$s, $\langle T_2\rangle \simeq 170\ \mu$s, and $\sigma_{T_1} = \sigma_{T_2} = 10\ \mu$s. We then vary the average coherence times across four orders of magnitude (the standard deviations are also scaled by the same factor), and simulate performance of the CE task analyzed in the main text; the resulting error rates are plotted in Supplementary Figure 6.

We note that for coherence times that are an order of magnitude shorter than the typical device coherence times, the CE task performance is essentially unaffected. In fact, even for very low coherence times of $\langle T_1\rangle \simeq 1\ \mu$s, around two orders of magnitude shorter than device lifetimes, a nontrivial I/O map is retained by the NISQRC algorithm and the considered instance of the CE task can still be performed (albeit with a testing error rate that now is marginally worse than that of single-step logistic regression). For even lower coherence times the I/O map will ultimately become trivial as errors uncorrelated with the input encoding start to overwhelm the dynamics of the system, and hence any outputs extracted from it.

Supplementary Note 8: IBM Device experiments under controlled delays

Fig. 4 of the main test shows the results of performing the CE task on the IBMQ device *ibm_algiers*. With the circuit we have employed, the total run time T_{run} approaches the average qubit T_1 times on this device. In principle, the NISQRC algorithm enables T_{run} to exceed T_1 indefinitely provided $T_1 > n_{\mathrm{M}}^0$ required for the specific instance of the CE task. However, due to limitations on the classical processing backend, the experiments are unable to be run for longer messages than $N = 20$ as of present, so that T_{run} cannot be increased naturally by increasing N.

In this Supplementary Note, we present the results of an experiment used to artificially lengthen the total circuit run time

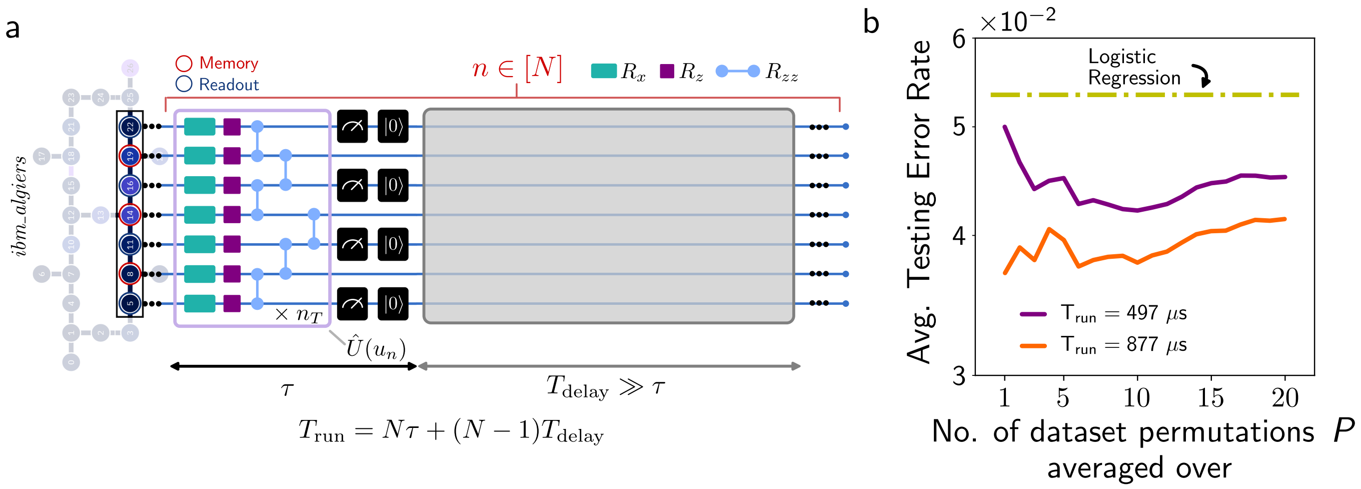

Supplementary Figure 7. **(a)** Experimental testing error rates for the CE task in the main text, Fig. 4 under the inclusion of controlled delays to increase total circuit run time much beyond individual qubit T_1 times, $T_{\rm run} \gg T_1$. Experiments are once again run on *ibm_algiers*, with mean qubit T_1 times of $\langle T_1 \rangle = 155\ \mu$s. **(b)** For comparison, we plot the testing error rate of logistic regression (yellow line).

$T_{\rm run}$ by introducing controlled delays to the circuit. The circuit schematic we implement is shown in Supplementary Figure 7(a), with the grey block indicating delays added after each set of gate applications, measurement, and reset operations, except after the final measurement. We consider delays that are typically much larger than the total time τ in each unit of evolution under NISQRC. We emphasize that during the delay time, the qubits forming the QRC can experience decay due to their finite lifetime. In the absence of delays, the circuit run time is $T_{\rm run} = 117\ \mu$s, as indicated in the main text. By introduce a delay of $T_{\rm delay} = 20\ \mu$s or $T_{\rm delay} = 40\ \mu$s per unit (significantly longer than the unit evolution time τ), the run time can be extended to $T_{\rm run} = 497\ \mu$s or $T_{\rm run} = 877\ \mu$s respectively; the latter is almost an order of magnitude larger than the mean $T_1 = 155\ \mu$s.

The testing error rate achieved is shown in Supplementary Figure 7(b). Here we show the performance cumulatively averaged over P permutations of the training and testing datasets, a standard cross-validation technique to remove fluctuations in performance when having access to only small datasets, and one we use for all results in the main text. We note that even with significantly longer run times, the device is able to beat logistic regression at the CE task. Increasing the delay from 20 μs to 40 μs per unit also does not significantly effect the performance, further highlighting the ability of NISQRC to overcome T_1 limitations on run time.

We note that the absolute performance shown in Supplementary Figure 7(b) is achieved with a larger $S \simeq 2^{16}$ than the largest value shown in Fig. 4 of the main text. The results in this Supplementary Note are calculated using data obtained several months after the data in Fig. 4 of the main text. The slight reduction in performance observed can be attributed to drift in the device over this time frame.

Supplementary Note 9: Lists of device parameters

Here we list device parameters used for producing Fig. 4(b) in the main text. All R_z gates are implemented as error-free virtual rotation z gates. Since all qubits used form a line, all the CNOT gate errors in the table are indexed by the qubits with small numbering in each control-target pair, therefore CNOT error is not applicable to the qubit 22. The averaged T_1, T_2 time over the three experiments are $T_1 \approx 124\ \mu$s and $T_2 \approx 91\ \mu$s.

Qubit	5	8	11	14	16	19	22
T_1 (μs)	159 ± 43	142 ± 29	144 ± 32	164 ± 20	160 ± 35	127 ± 38	147 ± 42
T_2 (μs)	96 ± 34	231 ± 30	32 ± 7	97 ± 4	68 ± 1	29 ± 7	163 ± 28
$\sqrt{X}$ error (%)	0.026 ± 0.006	0.043 ± 0.027	0.081 ± 0.060	0.037 ± 0.018	0.037 ± 0.005	0.050 ± 0.016	0.020 ± 0.005
CNOT error to next (%)	0.936 ± 0.571	1.028 ± 0.569	0.762 ± 0.228	6.850 ± 0.980	not reported	1.162 ± 0.251	N/A
Readout error (%)	0.746 ± 0.150	0.855 ± 0.093	1.412 ± 0.617	1.865 ± 0.528	26.292 ± 19.676	9.675 ± 0.789	1.129 ± 0.274
Readout length (μs)	$0.857\backslash 0.910$	$0.857\backslash 0.910$	$0.857\backslash 0.910$	$0.857\backslash 0.910$	$0.857\backslash 0.910$	$0.857\backslash 0.910$	$0.857\backslash 0.910$

Supplementary Table 1. Device parameters for connected QRC with mid-circuit measurement and deterministic reset (purple line in Fig. 4(b) of the main text). The calibrations to the CNOT gates between qubit 16 and qubit 19 are not successfully fitted, hence not reported by *ibqm_algiers* device. The re-calibration of readout length on July 14th, 2023, caused the pre$\backslash$post values $0.857\backslash 0.910$ μs, therefore the experiments for different shots S have different readout lengths.

Qubit	5	8	11	14	16	19	22
T_1 (μs)	63 ± 22	125 ± 29	115 ± 25	139 ± 32	90 ± 17	102 ± 19	120 ± 24
T_2 (μs)	93 ± 12	192 ± 52	26 ± 2	66 ± 16	9 ± 1	51 ± 3	146 ± 32
$\sqrt{X}$ error (%)	0.028 ± 0.005	0.020 ± 0.002	0.067 ± 0.025	0.028 ± 0.013	0.070 ± 0.010	0.030 ± 0.006	0.017 ± 0.001
CNOT error to next (%)	0.591 ± 0.074	2.255 ± 1.018	2.256 ± 1.139	1.655 ± 0.254	3.386 ± 0.491	0.848 ± 0.058	N/A
Readout error (%)	0.803 ± 0.108	0.771 ± 0.051	1.314 ± 0.349	3.561 ± 0.282	6.053 ± 1.643	2.546 ± 0.097	0.710 ± 0.084
Readout length (μs)	0.910	0.910	0.910	0.910	0.910	0.910	0.910

Supplementary Table 2. Device parameters for split QRC with mid-circuit measurement and deterministic reset (brown line in Fig. 4(b) of the main text).

Qubit	5	8	11	14	16	19	22
T_1 (μs)	63 ± 29	137 ± 15	118 ± 20	154 ± 26	114 ± 13	85 ± 25	133 ± 13
T_2 (μs)	75 ± 21	205 ± 39	26 ± 2	76 ± 8	10 ± 1	47 ± 7	159 ± 30
$\sqrt{X}$ error (%)	0.035 ± 0.010	0.018 ± 0.001	0.072 ± 0.054	0.017 ± 0.002	0.075 ± 0.019	0.030 ± 0.002	0.017 ± 0.002
CNOT error to next (%)	0.742 ± 0.321	0.954 ± 0.320	1.020 ± 0.264	1.564 ± 0.156	3.532 ± 0.605	0.840 ± 0.058	N/A
Readout error (%)	1.014 ± 0.464	0.770 ± 0.088	1.299 ± 0.183	3.438 ± 0.418	5.556 ± 1.982	2.473 ± 0.133	0.818 ± 0.142
Readout length (μs)	0.910	0.910	0.910	0.910	0.910	0.910	0.910

Supplementary Table 3. Device parameters for connected QRC with mid-circuit measurement, but without deterministic reset (green line in Fig. 4(b) of the main text).

SUPPLEMENTARY REFERENCES

[1] F. Hu, G. Angelatos, S. A. Khan, M. Vives, E. Türeci, L. Bello, G. E. Rowlands, G. J. Ribeill, and H. E. Türeci, Tackling sampling noise in physical systems for machine learning applications: Fundamental limits and eigentasks, Physical Review X **13**, 041020 (2023).

[2] J. Chen, H. I. Nurdin, and N. Yamamoto, Temporal Information Processing on Noisy Quantum Computers, Physical Review Applied **14**, 024065 (2020).

[3] B. Skinner, J. Ruhman, and A. Nahum, Measurement-induced phase transitions in the dynamics of entanglement, Physical Review X **9**, 031009 (2019).

[4] T. Yasuda, Y. Suzuki, T. Kubota, K. Nakajima, Q. Gao, W. Zhang, S. Shimono, H. I. Nurdin, and N. Yamamoto, Quantum reservoir computing with repeated measurements on superconducting devices, arXiv:2310.06706 [quant-ph] (2023).

[5] H. Jaeger and H. Haas, Harnessing Nonlinearity: Predicting Chaotic Systems and Saving Energy in Wireless Communication, Science **304**, 78 (2004).
